# Effects of Body Mass Index and Biochemical Lipid Levels on Reproductive Outcomes during An Intracytoplasmic Sperm Injection: A Retrospective Study

**DOI:** 10.22074/ijfs.2019.5614

**Published:** 2019-07-14

**Authors:** Luisa Maria Di Gregorio, Elisa Zambrotta, Federica Di Guardo, Ferdinando Antonio Gulino F, Giulia Musmeci, Stella Capriglione, Roberto Angioli, Marco Palumbo

**Affiliations:** 1 Department of General Surgery and Medical Surgical Specialties, University of Catania, Catania, Italy; 2 Department of Pharmaceutical Sciences, University of Catania, Catania, Italy; 3 Department of Obstetrics and Gynaecology, Campus Bio-Medico, Rome, Italy

**Keywords:** Body Mass Index, Infertility, *In vitro* Fertilization, Metabolic Diseases

## Abstract

**Background:**

The aim of this study was to evaluate the impact of body mass index (BMI) and lipid profile on repro- ductive outcomes of women undergoing intracytoplasmic sperm injection (ICSI) cycles.

**Materials and Methods:**

This retrospective observational study was conducted in the Center of Human Reproductive Physiopathology of University of Catania between April 2017 and March 2018 and enrolled 114 couples undergoing ICSI. Levels of total cholesterol, low-density lipoprotein-cholesterol (LDL-c), high-density lipoprotein-cholesterol (HDL-c) and triglycerides were determinate and, according to the BMI, samples were divided into the following groups: group A (BMI: 18.5-24.9 kg/m^2^); group B (BMI: 25-29.9 kg/m^2^); and group C (BMI >30 kg/m^2^). BMI and lipid profile associations with the number of oocytes and embryos retrieved, the oocytes and embryo quality, the fer- tilization rate as well as the percentage of miscarriages and pregnancies, were assessed. The statistical analysis was performed using Shapiro-Wilk test, analysis of variance (ANOVA) and Kruskal -Wallis method.

**Results:**

Fertilization and pregnancy rates were lower in women with BMI>30 than in women with BMI: 25-29.9 and BMI: 18.5-24.9, despite the not altered levels of lipoprotein.

**Conclusion:**

Our results demonstrated that an excess of adipose tissue in women undergoing ICSI was not directly related with altered biochemical lipid values. However, overweight and obese patients showed poor fertilization and pregnancy rate despite the not altered values of lipoprotein.

## Introduction

Variations in lipid asset, body mass index (BMI) and abdominal adipose tissue affect the female reproductive system compromising ovulatory cycle, oocytes and embryo quality. Obesity is associated with hyperinsulinemia and insulin resistance causing an increased hormonal ovarian production and a reduced synthesis of sex hormone-binding globulin (SHBG), with subsequent hyperandrogenism. A peripheral conversion of ovarian and adrenal androgens determinates abnormal secretions of gonadotropin-releasing hormone (GnRH), a reduced peak of luteinizing hormone (LH) and metabolites of progesterone (1). These factors are in favour of development of anovulatory cycles, oligomenorrhea/ amenorrhea, atypical follicular recruiting, poor oocytes quality, endometrial development failure and impaired corpus luteum function (2). The excess of abdominal adipose tissue induces the release of non-esterified acids causing a lipid increase in muscle and liver followed by dyslipidaemia (3). Dyslipidaemia is characterized by elevated plasma levels of triglycerides and low-density lipoprotein-cholesterol (LDL-c), small catabolism of apolipoprotein B (Apo- B) and increased degradation of high-density lipoprotein (HDL)-apoa-I (4). It was shown that obesity and its complications have negative consequences for oocyte quality, fertilization rate, embryo development and pregnancy rate (5).

Despite this fact, recent studies, did not report differences in oocyte maturity in function of BMI variation (6); the same cannot be said about the fertilization rate that seems to reduce proportionally with the increasing BMI; in particular, a 45% reduction in fertilization rate was identified in women with BMI>30 (7, 8). The effects of female obesity on embryo quality were also investigated during the last 10 years. Results indicated that embryo quality influences the number of discarded embryos, the number of frozen embryos and the rate of embryo used with a mean grade of embryos significantly lower in obese patients (9). Finally, it was shown that pregnancy and live birth rate drastically decrease when BMI increases; on the other hand, the pregnancy rate was not compromised in obese women receiving high quality heterologous oocytes suggesting that high BMI acted on multiple levels of reproductive process, including oocytes and embryos (10). Finally, no adverse effect of BMI on implantation and pregnancy rate was found in obese women receiving donor oocytes, supporting the hypothesis that BMI does not influence endometrium receptivity while oocytes and embryos of poor quality can produce poor reproductive results.

The aim of our study was to evaluate possible correlations between variations in BMI, triglycerides, HDL-c, LDL-c, and total cholesterol levels and ovarian response (in terms of oocytes and embryos retrieved, oocytes and embryo quality, fertilization rate and percentage of miscarriages and pregnancies) in women undergoing intracytoplasmic sperm injection (ICSI) cycles.

## Materials and Methods

We conducted a retrospective observational study on 114 couples undergoing ICSI who referred to our Human Reproductive Physiopathology Centre between April 2017 and March 2018. Each couple, before entering the study, subscribed an informed consent, and anonymity was preserved. The study protocol conformed to the ethical guidelines of the Helsinki Declaration (as revised in Tokyo 2004) and was approved by the Local Research Ethics Committee. The enrolled couples underwent ICSI for idiopathic causes after a history of primary or secondary infertility from almost one year, and met the following criteria. 

Inclusion criteria: i. Female age between 18-42 years, ii. Primary or secondary infertility for not less than 12 months, iii. ICSI techniques, iv. Negative results for Hepatitis B Australia Antigen (HbsAg), Hepatitis C Virus (HCV), Human Immunodeficiency Virus (HIV) for the male and the female, v. No exposure to toxic agents reported by the couples, vi. Female BMI<35, vii. Normal semen parameters, and viii. Follicle stimulating hormone (FSH) <15 IU/mL.

Exclusion criteria: i. Female age <18 or >42 years, ii. Primary or secondary infertility for less than 12 months, iii. Assisted reproductive techniques (ART) different from ICSI, iv. Positive results for HbsAg, HCV, HIV for the male and the female, v. Exposure to toxic agents reported by the couples, vii. Female BMI >35, vii. severe oligozoospermia, severe asthenozoospermia, and/or azoospermia, viii. FSH >15 IU/mL.

Following obtaining the signed informed consent, FSH, LH, estrogen (E2) and prolactin (PRL) blood test were done on the 3rd day of menstrual cycle. PRL samples were collected three to four hours after waking up in the morning by taking three samples with 20 minutes intervals after correcting the position of the vein catheter. Moreover, blood tests were performed in order to determinate plasma concentrations of total cholesterol, LDL-c, HDL-c and triglycerides. The blood samples were obtained before starting ovarian stimulation by using separated lipoproteins, by electrophoresis or precipitation method. Finally, weight and height of the patients were recorded. According to the BMI, the samples were divided into the following groups: group A: patients with BMI of 18.5-24.9 kg/m^2^ (45.6%); group B: patients with BMI of 25-29.9 kg/m^2^ (32.4%); and group C: patients with BMI greater than 30 kg/m^2^ (22%). Woman who participated in the present study, had been treated with gonadotropin-releasing hormone (GnRH) agonist long protocol using leuprorelin acetate or triptorelin acetate and recombinant FSH was injected 14 days later. E2 level dosage and ultrasound control were performed three times a week after starting recombinant FSH therapy to monitor the follicular growth. When the E2 level and the follicular dimensions were adequate, human chorionic gonadotropin (hCG) triggering was performed using chorionic gonadotropin. All the pick-ups were performed 36- 38 hours after the hCG triggering. Retrieved oocytes were inseminated by ICSI and thus, the zygotes were observed at their maturation by biologist. Embryos were transferred 48-72 hours after the pick-ups. Variable considered in our study were as follows: number of retrieved oocytes and obtained embryos as well as oocytes’ and embryo quality using respectively “Pronuclear Scoring System” and “Day 3 Scoring”.

The pronuclear scoring system was used with the aim of detecting the pronuclear stage. The evaluation began about 16-20 hours after ICSI. Oocytes were examined on the heated stage of an inverted microscope equipped with Hoffman modulation contrast (200 magnification). Normally fertilized oocytes presented two clearly distinct pronuclei (2PN) and two polar bodies. This pattern correlated with increased embryo competence. The presence of one pronucleus (1PN) might be a result of errors in the fertilization process due to the asynchrony in formation/fusion of pronucleus. The formation of three different pronuclei might be due to an altered fertilization process determining a triploid zygote. The “Day 3 Scoring” system evaluates the morphological appearance of embryo on day 3. Embryos were assessed using a scoring system graded 1 to 5 according to morphology, which took into account cell number, evenness of cell division and degree of fragmentation. In the following lines, the characteristics of each grade are explained. Grade 1: 8 cells, <10% fragmentation, good cell-cell contact, absence of multinucleated blastomers, grade 2: 8 cells, 10-20% fragmentation or lacking good cell-cell contact, absence of multinucleated blastomers, grade 3: 6-7 cells or 8 cells with 20% fragmentation or uneven blastomer size, absence of multinucleated blastomers, grade 4: >8 cells or 4-6 cells or 8 cells with >20% fragmentation or uneven blastomer size or multinucleated blastomers, grade 5: <4 cells or grossly fragmented or with half of the blastomers being multinucleated. 

Moreover, our study evaluated also the fertilization and rate and percentage of miscarriages as well as clinical pregnancies detected first by β-hCG blood test after 14 days from embryo transfer and then by transvaginal ultrasound visualization.

### Statistical analysis

The primary endpoint was the establishment of a correlation between variations of BMI, triglycerides, HDL- c, LDL-c, and total cholesterol and ovarian response (in terms of oocytes and embryos retrieved, oocytes and embryo quality, fertilization rate and percentage of miscarriages and pregnancies) in ICSI cycles. The Shapiro-Wilk test was implemented to test the normally distribution of the variables. The level of significance of normality test for all variables was alfa=0.05. Analysis of variance (ANOVA) was applied to compare normally distributed variables. Variables with free distributions were analyzed using Kruskal-Wallis method. Normally distributed continuous data were presented as mean ± SD, while categorical data were presented as number (n) and percentage (%). Non-normally distributed data were presented as median (range).

## Results

We demonstrated that normal weight patients (group A) and overweight patients (group B) had mean HDL-c levels, with parameters included within the normal range. Significantly high values were found for obese patients (group C) with a mean value of 45.3 mg/dl (SD: 21.68, P=0.0032) for HDL-c which was close to the inferior normal range and the mean value for triglycerides was 101.5 mg/dl (SD: 23.7, P=0.00001, Table 1). All the patients were treated with a long stimulation protocol. Also, we found a reduction in the mean value of retrieved oocytes (mean ± SD: 3.0 ± 3.16, P=0.00001) as well as fertilized oocytes (mean ± SD: 1.25 ± 1.50, P=0.00001) in group C respect in group A and B (Table 2). In group A, 106 embryos were obtained from a total of 122 fertilized oocytes (86.8%), with a mean value of 1.81 ± 1.15 (mean ± SD, P=0.25). In group B, 71 embryos were obtained from a total of 90 fertilized oocytes (78.8%), with a mean value of 1.94 ± 1.06 (mean ± SD, P=0.71). In group C, 18 embryos were obtained from a total of 25 fertilized oocytes (73%), with a mean value of 1.00 ± 1.15 (mean ± SD, P=0.0036, Table 2). The fertilization rate was 70.52% [confidence interval (CI): 32-82% for 122 fertilized oocytes] in group A, 70.9% (CI: 30-84.2% for 90 fertilized oocytes) in group B and 62.5% (CI: 24.3-73.2% for 25 fertilized oocytes) in group C, with similar value between normal-weight and overweight patients while it was slightly reduced in obese women. Data concerning oocyte maturation and zygotes pronuclear number for each group are reported in Table 3. Data referred to embryo grade of maturation descripted by Day 3 Scoring System are reported in Table 4. Fourteen days after embryo-transfer, blood levels of β-hCG were measured. Blood β-hCG was negative for 93 patients (80.9%, CI: 35-94.4%) and positive in 22 patients (19.1%, CI: 9.3-37.8%). Of these 22 patients, 17 were from group A, 3 were from group B, and the remaining 2 were from group C. We observed 3 biochemical miscarriages in group A, 6 clinical miscarriages equally distributed among group A, B and C, 12 pregnancies in group A and 1 pregnancy in group B and 0 pregnancy in group C (Table 5).

**Table 1:** Levels of blood lipids


Variables (mg/dl)	Group A		Group B		Group C	
	Mean ± SD	P value	Mean ± SD	P value	Mean ± SD	P value

Total cholesterol	183 ± 33.43	0.23	178 ± 23.89	0.12	165 ± 21.79	0.071
LDL	110 ± 31.14	0.14	101 ± 25.71	0.13	101 ± 23.89	0.16
HDL	64.8 ± 17.49	0.16	60.4 ± 10.34	0.13	45.3 ± 21.68	0.0032
Triglycerides	68.1 ± 19.30	0.08	62.9 ± 21.08	0.24	101.5 ± 23.7	0.00001


P<0.05 were considered significant. LDL; Low-density lipoprotein and HDL; High-density lipoprotein.

**Table 2:** Number of retrived oocytes, fertilized oocytes and embryo obtained from each group


Variables	Group A		Group B		Group C	
	Mean ± SD	P value	Mean ± SD	P value	Mean ± SD	P value

Retrived oocytes	7.88 ± 5.99	0.32	6.25 ± 4.15	0.13	3.0 ± 3.16	0.00001
Fertilized oocytes	2.97 ± 1.36	0.22	2.19 ± 1.32	0.003	1.25 ± 1.50	0.00001
Total embryos	1.81 ± 1.15	0.25	1.94 ± 1.06	0.71	1.00 ± 1.15	0.0036


P value are significant if <0.05.

**Table 3:** Number and percentage of oocytes in stage of maturation and zygotes pronuclear number


Oocytes/Zigotes	Median	Group A (%)	Group B (%)	Group C (%)

M1	18 (20-3)	11.5 (20/173)	14 (18/127)	7.5 (3/40)
M2	95 (136-34)	78.6 (136/173)	75 (95/127)	85 (34/40)
GV	12 (13-3)	7 (12/173)	10 (13/127)	7.5 (3/40)
DEG	1 (5-0)	2.9 (5/173)	1 (1/127)	0 (0/40)
PN1	5 (7-0)	4.1 (5/122)	7.7 (7/90)	0 (0/25)
PN2	83 (20-117)	95.9 (117/122)	92.3 (83/90)	80 (20/25)
PN3	0 (5-0)	0 (0/122)	0 (0/90)	25 (5/25)


Median (range) is used to indicate the middle value of zygotes in a determinate stage of maturation and with a determinate pronuclear number. Percentage is used to indicate the number of zygotes in a determinate stage of maturation and the percentage of zygote with a determinate pronuclear number in each group. M1; Immature retrieved oocytes in metaphase I, M2; Mature retrieved oocytes in metaphase II, GV; Germinal vesicular, DEG; Degenerated oocytes, and PN1-PN2-PN3; Zygotes with 1 pronucleus, 2 pronuclei or 3 pronuclei.

**Table 4:** Number and percentage of embryo descripted by day 3 scoring system


Embryos	Median	Group A (%)	Group B (%)	Group C (%)

G1	16 (42-0)	39.6 (42/106)	22.5 (16/71)	0 (0/18)
G2	25 (46-6)	43.4 (46/106)	35.0 (25/71)	33.3 (6/18)
G3	14 (16-5)	15.1 (16/106)	20.0 (14/71)	27.7 (5/18)
G4	2 (5-0)	1.9 (2/106)	7.0 (5/71)	0 (0/18)
G5	0 (2-0)	0 (0/106)	2.8 (2/71)	0 (0/18)
DEG	7 (9-0)	0 (0/106)	12.7 (9/71)	40 (7/18)


Median (range) is used to indicate the middle value of embryo in a determinate stage of maturation percentage is used to indicate the stage of maturation of embryo catalogued by day 3 scoring system in each group. G1; Grade 1 embryo according day 3 scoring system, G2; Grade 2 embryo according day 3 scoring system, G3; Grade 3 embryo according day 3 scoring system, G4; Grade 4 embryo according day 3 scoring system, G5; Grade 5 embryo according day 3 scoring system, and DEG; Degenerated embryo.

**Table 5:** Number and percentage of positive βhCG, biochemical miscarriages, clinical miscarriages and clinical pregnancies


Embryos	Median	Group A (%)	Group B (%)	Group C (%)

Positive βhCG	3 (17-2)	16 (17/106)	4.2 (3/71)	11.1 (2/18)
Biochemical miscarriages	0 (3-0)	1.8 (3/106)	0 (0/71)	0 (0/18)
Clinical miscarriages	2 (2-2)	1.8 (2/106)	2.8 (2/71)	11.1 (2/18)
Clinical pregnancies	1 (12-0)	1.9 (12/106)	1.4 (1/71)	0 (0/18)


βhCG; Beta human chorionic gonadotropin. Median (range) is used to indicate the middle value of positive βhCG, biochemical miscarriages, clinical miscarriages and clinical pregnancies. Percentage is used to indicate the percentage of positive βhCG, biochemical miscarriages, clinical miscarriages and clinical pregnancies for each group.

## Discussion

The present study aimed to investigate the relationship between obesity and lipid variations and to determinate their association with ovarian response in terms of oocyte maturation, fertilization rate, embryo quality and pregnancy rate in women undergoing ICSI. Our literature survey demonstrated that obesity is strongly associated with metabolic syndrome (11), polycystic ovary syndrome (PCOS) (12) and dyslipidaemia (13). These conditions are characterized by hyperinsulinemia (14) and insulin resistance (13). High BMI causes ovulatory dysfunctions, anovulatory cycles, infertility (15) and hyperandrogenism by the reduction of SHBG as well as by hyperinsulinemia (16). Obesity and its complications were shown in both animal and human studies, to have negative consequences in oocyte quality, fertilization rate, embryo development and pregnancy rate (5). With respect to oocyte quality and maturation, our study showed a similar percentage of M2 oocytes in obese patients (85%) compared to overweight (75%) and normal-weight women (78.6%). This result is in accordance with the study of Shalom-Paz et al. (6) that did not report any BMI influence on *in vitro* maturation of oocytes in PCOS women. Despite the normal values of cholesterol in our samples, we found a lower fertilization rate in woman with BMI >30 compared to the other two groups, since the metabolic disorders related to obesity induced ovarian resistance towards the action of high-dose gonadotropins (17). The same result was obtained in the study of van Swieten et al. (7) that identified a 45% reduction in fertilization rate in women with BMI>30. On the other hand, Salha et al. (8) found that fertilization rates were 26.6% in patients with BMI .26 and 37.1% in patients within normal BMI range.

Considering the effect of obesity on embryo quality, Metwally et al. (9) after assessing the embryo quality on the 2^nd^ day following the oocyte pick-ups, investigated the shape of blastomeres, the cytoplasm structure and the degree of fragmentation, and they demonstrated that the mean grade of embryo quality is significantly lower in obese patients. Our data confirmed that obesity alters the quality and the number of embryos obtained with a mean value of 2 embryos in normal weight patients with normal lipid profile while averagely 1 embryo was obtained from patients with BMI>30 and plasma lipids concentrations near limits. We found that the percentage of embryos with morphological characteristics for pregnancy, decreased inversely proportional to BMI while the percentage of embryos with morphological anomalies increased proportional to BMI.

Finally, results of our study reported a rate of 19.1% for β-hCG positivity, with greater success among normal- weight, compared to overweight and obese patients. The pregnancy rate was 85.7% (12/14 pregnancies) in group A, and 14.3% (2/14 pregnancies) in group B, but no conception in group C. Consistent with our findings, as far as the pregnancy and live birth rate is concerned, Luke et al. (10) reported that pregnancy and live birth rate drastically decreased when BMI increased, while it seems likely that pregnancy rate was not influenced by obesity when women received high-quality heterologous oocytes. The correlation between obesity and pregnancy rate in ART was demonstrated in 3 large studies which revealed a reduced probability of pregnancy which was directly proportional to high BMI (18-20). Moreover, obesity causes a reduction of fertility in women undergoing ART cycles and in those who conceive spontaneously (21) demonstrating that high BMI adversely affects oocytes and embryo quality as reflected by a reduction of pregnancy rates (22).

## Conclusion

Our study demonstrated that an excess of adipose tissue in women undergoing ICSI was not directly related with altered values of lipoprotein taken in consideration in our study. Overweight and obese patients (BMI: 25-34) showed poor fertilization and pregnancy rates despite the not altered levels of lipoprotein. Strengths of our work were the accurate collection of data on oocyte and embryo quality, as well as the careful processing of collected values. However, as our study was conducted in a small population, further research should be done to better understand the pathogenic mechanisms underlying poor reproductive outcomes in obese and overweight women. Finally, we believe that young women of reproductive age should be appropriately advised about the negative effects of obesity and insulin resistance on fertility, in order to perform some lifestyle modification.
